# Purpose under pressure: how calling, recovery, emotional regulation and flow shape elite athletic performance

**DOI:** 10.3389/fpsyg.2026.1894992

**Published:** 2026-07-22

**Authors:** Liliana Pitacho, João Pedro Cordeiro

**Affiliations:** 1 Escola Superior de Ciências Empresariais (ESCE), Instituto Politécnico de Setúbal (IPS), Setúbal, Portugal; 2 Centro de Investigação em Qualidade de Vida – CIEQV, Santarém, Portugal

**Keywords:** athletic performance, competitive anxiety, flow state, psychological resources, recovery, self-confidence, sport calling

## Abstract

**Introduction:**

Athletic performance in professional roller hockey is influenced not only by physical, technical, and tactical factors, but also by psychological resources that help athletes regulate pressure, sustain recovery, and enter optimal performance states. This study examined sport calling as a relatively stable psychological resource for understanding performance under pressure in professional roller hockey. Drawing on a demands–resources perspective applied to sport, we tested whether baseline sport calling was associated with competitive performance directly and indirectly through perceived well-being/recovery, pre-competitive psychological states, and flow.

**Methods:**

A longitudinal field design with repeated measures was conducted during a four-day knockout tournament involving 80 male professional roller hockey players.

**Results:**

Linear mixed models indicated that baseline sport calling was positively associated with competitive performance, flow, and self-confidence, and negatively associated with poorer perceived recovery, cognitive anxiety, and somatic anxiety across the tournament. Exploratory time-specific mediation analyses further suggested that the association between sport calling and performance was consistent with an indirect pathway involving perceived recovery, self-confidence, and flow at the first and second competitive stages.

**Discussion:**

These findings suggest that sport calling may be understood as a motivational and identity-based resource associated with more adaptive psychological functioning and competitive performance under pressure. The study contributes to sport psychology by positioning calling as a promising construct for understanding performance, recovery, and optimal experience in elite sport contexts.

## 1 Introduction

From a traditional perspective, athletic performance in high-performance contexts has been understood as the result of the interaction between biophysical, technical, and tactical factors. However, more recent research has broadened this view by introducing psychological resources as a fourth central element in the explanation of athletic performance. This shift is particularly relevant in elite sport, where the margin between success and failure is often narrow and where small variations in recovery, emotional states, self-confidence, or optimal functioning may distinguish average from superior performances. Thus, athletic performance should be understood as the result of a complex interaction between physiological capacity, technical-tactical skills, psychological readiness, and emotional and recovery-related self-regulation processes (Gershgoren et al., 2023; Gouttebarge et al., 2019; Henriksen et al., 2020; Kuettel and Larsen, 2020; Reardon et al., 2019; Rice et al., 2016; Schinke et al., 2018; Silva et al., 2026).

Understanding athletic performance requires a multidimensional perspective that considers not only risk factors, such as anxiety, fatigue, or insufficient recovery, but also adaptive resources, including purpose, self-confidence, and flow experience. This perspective can be framed through an adaptation of the Job Demands–Resources model (Bakker and Demerouti, 2007) to sport, assuming that performance results from the balance between competitive demands and athletes’ available resources (Balk et al., 2020). In elite sport, demands include physical intensity, time pressure, tactical uncertainty, evaluative exposure, rapid decision-making, and emotional load. Highly demanding contexts also involve strong attentional requirements, perceived control, and environmental uncertainty, making psychological self-regulation central to performance (Hsu, 2026). In team sports, competition imposes a high mental load through continuous concentration, contextual monitoring, interpretation of teammates’ and opponents’ movements, and rapid decisions (Moen et al., 2021). Competition is also emotionally activating, making emotional regulation crucial under pressure (Buchanan and Janelle, 2022; Gershgoren et al., 2023). When demands exceed resources, athletes may experience anxiety, fatigue, and performance deterioration; conversely, robust resources may help athletes appraise demands as motivating challenges and may be associated with stronger self-regulation and persistence (Bakker and Demerouti, 2007; Balk et al., 2020).

Within this framework, sport calling is used in the present study as a psychological construct rather than as a colloquial synonym for passion, preference, or vocation. Drawing on contemporary calling theory and its extension to the sport domain, sport calling can be conceptualized as an internally driven psychological orientation reflecting a strong alignment between identity, purpose, and engagement in sport. Calling has been described as a multidimensional orientation grounded in meaningful and persistent passion, identity centrality, purpose, commitment, engagement, and willingness to sacrifice (Dobrow and Tosti-Kharas, 2011; Dobrow et al., 2023; Duffy et al., 2014; Pitacho and Cordeiro, 2023). Applied to sport, calling may therefore be understood as a deeply embedded person–activity relationship through which athletes experience sport as a central source of identity, meaning, purpose, personal fulfilment, and sustained commitment. Although still underexplored in sport, this context is especially relevant for the emergence of calling. Ronkainen et al. (2018) argued that calling may help explain meaningful lives and careers in sport. Elite athletes often invest intensively in their discipline from an early age, organize their lives around training and competition, and strongly identify with the athletic role. Consequently, sport may become a central domain through which athletes construct meaning and personal value.

Although calling should not be understood as entirely fixed, the literature conceptualizes it as a relatively stable psychological orientation, closer to a trait than a transient state (Dobrow and Tosti-Kharas, 2011; Vianello et al., 2022). By giving meaning to competition and sustaining engagement in demanding contexts, sport calling may represent a stable psychological resource that protects against competitive strain and supports self-confidence, perceived recovery, and optimal functioning states such as flow. Thus, within a Demands-Resources logic, calling may be relevant for understanding why some athletes respond to competitive demands with more adaptive performance responses.

In this sense, the following hypothesis is proposed:

*H1*. Sport calling will positively associate with competitive performance across the tournament, such that athletes reporting higher levels of calling before the competition will show higher performance ratings across competitive moments.

The relationship between sport calling and competitive performance can be supported by literature describing calling as a motivational, identity-based, and behavioral resource. Calling is not merely a subjective preference or sense of meaning, but a psychological orientation with behavioral implications (Duffy et al., 2018). Several studies have demonstrated positive relationships between calling and performance, either directly or indirectly (e.g., Vianello et al., 2022; Yu, 2022). These findings suggest that calling may translate into observable performance behaviors through greater initiative, self-regulation, and adaptation to task demands. This relationship is reinforced when objective indicators of success are considered. In career research, objective success is typically operationalized through external indicators such as salary, promotions, and occupational status. Some studies identify calling as a predictor of these outcomes, showing that individuals with higher levels of calling are more likely to achieve higher status, greater remuneration, and more promotions than those with a more instrumental orientation (Abele and Spurk, 2009; Cho and Jiang, 2022; Ng et al., 2005).

The following hypothesis is therefore proposed:

*H2*. Sport calling will positively associate with flow across the tournament, such that athletes reporting higher levels of calling before the competition will experience higher levels of flow across competitive moments.

Flow is used in the present study in its established psychological meaning, rather than in its everyday sense of movement, continuity, or transfer of energy. Accordingly, in the context of this manuscript, flow refers to a state of optimal subjective experience during performance, in which athletes perceive their actions as fluid, focused, controlled, and intrinsically rewarding (Swann et al., 2012). In this theoretical tradition Flow is widely described as an optimal psychological state characterized by deep concentration, action–awareness merging, perceived control, clear goals, immediate feedback, altered perception of time, and intrinsic reward (Csikszentmihalyi, 1990; Jackson and Marsh, 1996). In sport, flow has been associated with optimal functioning, high-quality performance, confidence, automaticity, and the ease with which sport-specific actions are executed (Antonini Philippe et al., 2022). Recent studies have also emphasized that flow comprises cognitive, physiological, and affective components, and that emotions play a crucial role in the preparation, entry, maintenance, and termination of this state (Antonini Philippe et al., 2022; Biasutti and Philippe, 2023). Thus, calling may constitute a relevant antecedent of this optimal state, insofar as both constructs share a strong component of meaning, engagement, and absorption in the activity (Csikszentmihalyi, 1990; Dobrow and Tosti-Kharas, 2011; Pitacho and Cordeiro, 2023).

*H3*. Sport calling will positively associate with perceived well-being/recovery across the tournament.

Maintaining the perspective of sport calling as a relatively stable psychological resource, it may promote wellbeing and recovery by shaping how athletes interpret, tolerate, and regulate competitive demands. Work as Calling Theory proposes that experiencing an activity as a calling enhances meaning, satisfaction, and wellbeing, particularly when individuals translate this purpose into functional and self-regulated engagement (Duffy et al., 2018). Similarly, vocational research shows that perceiving a calling is positively associated with life satisfaction and wellbeing through mechanisms such as meaning in life, motivation, and the ability to live one’s calling effectively (Duffy et al., 2017). Applied to elite sport, this suggests that athletes who perceive sport as a central source of purpose and identity may cope more effectively with pressure and emotional demands because they possess a psychological framework that gives meaning to effort and supports adaptive self-regulation. This is especially relevant because recovery is now understood as a multidimensional process involving physiological restoration, psychological readiness, fatigue, stress, sleep, and adaptation to training loads. Silva et al. (2026) emphasize that recovery should be understood integrative, considering emotional regulation, biological readiness, and sport-specific demands. Thus, calling may be associated with more favorable recovery resources by reinforcing restorative behaviors, supporting emotional regulation, and sustaining purpose under pressure and fatigue.

Following this adaptive function, sport calling may also constitute an important antecedent of pre-competitive psychological states, particularly self-confidence and competitive anxiety. Self-confidence refers to athletes’ belief in their ability to respond effectively to competitive demands and is consistently identified as a key psychological resource in performance contexts, reducing perceived threat, facilitating task focus, and supporting adaptive coping strategies (Bandura, 1997; Woodman and Hardy, 2003; Craft et al., 2003). In contrast, cognitive anxiety involves worries and doubts about performance, whereas somatic anxiety reflects perceived physiological activation associated with competition (Martens et al., 1990). Recent research shows that self-confidence is linked to greater psychological readiness and more favorable outcomes, whereas anxiety, especially when interpreted as threatening, may impair attention, decision-making, and emotional regulation (López-Hernández et al., 2026; Peng et al., 2025). Athletes who experience sport as a calling are more likely to perceive competition as an opportunity for identity expression and purpose fulfilment rather than as an externally evaluative situation. This alignment between identity, meaning, and action may strengthen perceptions of competence and control, enhancing self-confidence while reducing threat-based appraisals and maladaptive anxiety responses. Thus, calling may be understood as a motivational and identity-based resources associated with athletes’ tendency to appraise competitive pressure less as a threat and more as a challenge. Based on this rationale, the following hypotheses are proposed:

*H4*. Baseline sport calling will positively associate with self-confidence across the tournament, such that athletes reporting higher levels of calling before the competition will show higher self-confidence across competitive moments.

*H5*. Baseline sport calling will negatively associate with cognitive anxiety across the tournament, such that athletes reporting higher levels of calling before the competition will experience lower cognitive anxiety across competitive moments.

*H6*. Baseline sport calling will negatively associate with somatic anxiety across the tournament, such that athletes reporting higher levels of calling before the competition will experience lower somatic anxiety across competitive moments.

Considering the relationships previously proposed, it is theoretically coherent to advance an integrative model in which sport calling is examined not only as a factor associated with performance, but also as a distal resource linked with recovery-related, emotional, and experiential mechanisms proximal to competition. If calling gives meaning to competitive demands, supports self-regulation under pressure, and sustains coherence between identity, purpose, and action, its association with performance may be partly reflected in an adaptive sequence of functioning. Athletes with higher sport calling are expected to report more favorable perceptions of wellbeing and recovery, creating a stronger psychophysiological basis for competition. Better perceived recovery should promote more adaptive pre-competitive states, reflected in higher self-confidence and lower cognitive and somatic anxiety (López-Hernández et al., 2026; Martens et al., 1990). Together, these states facilitate entry into flow, which depends on attentional focus, perceived control, challenge–skill balance, and task absorption (Domínguez-González et al., 2024; Koehn, 2013; Pitacho, 2009). Flow then becomes the mechanism most proximal to effective execution, translating psychological resources into fluid and contextually adjusted sport action (Antonini Philippe et al., 2022; Swann et al., 2012). Based on this rationale, the following hypothesis is proposed:

*H7*. The relationship between baseline sport calling and competitive performance will be serially mediated by perceived well-being/recovery, pre-competitive psychological states, and flow. Specifically, athletes reporting higher sport calling before the competition are expected to report more favorable perceived recovery, which in turn is expected to be associated with more adaptive pre-competitive states, namely higher self-confidence and lower cognitive and somatic anxiety. These states are expected to be associated with higher flow and, ultimately, better competitive performance.

## 2 Methods

This study adopted a longitudinal field design with repeated measures embedded in a naturalistic knockout competition setting. The design combined a baseline assessment of relatively stable individual characteristics with repeated assessments of state-based psychological and performance-related variables across successive competitive stages. Given the progressive elimination structure of the tournament, the study involved an unbalanced repeated-measures design, in which the number of observations decreased over time as teams were eliminated. This design was appropriate for examining within-athlete changes and associations among psychological states, perceived recovery, flow, and performance under ecologically valid competitive conditions.

The study was conducted in accordance with the ethical principles of the Declaration of Helsinki. All athletes were informed about the aims of the study, the voluntary nature of participation, and the confidentiality of their responses. Written informed consent was obtained prior to data collection.

### 2.1 Participants

Participants were 80 male professional roller hockey players from the Portuguese National First Division. The athletes were between 18 and 33 years old (*M* = 24.36, SD = 3.80). At the time of data collection, all participants represented one of the eight highest-ranked teams in the national championship and took part in the final knockout competition analyzed in this study.

### 2.2 Procedures

Data were collected during a four-day elimination tournament involving the eight highest-ranked teams, at that stage of the season, from the Portuguese National First Division Roller Hockey Championship. All teams were accommodated in the same hotel near the sports venue, allowing standardized data collection across teams and competition days.

The tournament followed a knockout format. On Day 1, all eight teams competed in four matches. Day 2 was a rest day. On Day 3, the four winning teams competed in the semi-finals, and on Day 4, the two remaining teams played the final.

Before the competition (Day 0), athletes completed a baseline questionnaire including sociodemographic information and the sport-calling measure. Each athlete received an identification code to match responses across assessment moments while preserving anonymity.

On each competition day, data collection followed the same sequence. In the morning, before breakfast, athletes completed the Hooper Index to assess perceived wellbeing/recovery. Approximately 2 h before each match, they completed the CSAI-2 to assess cognitive anxiety, somatic anxiety, and self-confidence. Immediately after each match, and before showering, athletes completed the Flow State Scale-2 regarding their match experience. This procedure was repeated for all athletes whose teams remained in the tournament.

Because of the elimination format, the number of participants decreased across time points. Thus, all athletes were assessed on Day 1, semi-finalists on Day 3, and finalists on Day 4, allowing repeated psychological and performance-related assessments under real competitive conditions.

### 2.3 Instruments

#### 2.3.1 Calling scale

Calling was assessed once at baseline using a 10-item adapted version of the calling scale from the Work Orientation Questionnaire (WOQ; Pitacho et al., 2019). The original WOQ was developed to assess individuals’ orientations toward work and includes three independent scales: calling, career, and job. In the present study, 10 items from the original calling scale were retained and adapted to the sport context to capture the extent to which athletes perceived their sport participation as a source of meaning, purpose, personal fulfilment, and identity. Unlike the state-based variables assessed across the competitive moments, calling was conceptualized as a relatively stable motivational orientation and was therefore measured only once at baseline. Items were rated on a Likert-type scale ranging from 1 to 10, with higher scores indicating stronger sport calling. A global calling score was computed by averaging the 10 items. The adapted scale showed excellent internal consistency in the present sample, with Cronbach’s alpha of 0.954.

#### 2.3.2 Competitive state anxiety

Competitive state anxiety was measured using the Competitive State Anxiety Inventory-2 (CSAI-2; Martens et al., 1990). The instrument includes 27 items distributed across three subscales: cognitive anxiety, somatic anxiety, and self-confidence, each comprising nine items. Items are rated on a 4-point Likert-type scale ranging from 1 (not at all) to 4 (very much so). Cognitive anxiety captures athletes’ worries and negative performance-related expectations, somatic anxiety reflects perceived physiological activation, and self-confidence assesses athletes’ perceived confidence in their ability to perform successfully. Subscale scores were calculated by averaging the respective items, with higher scores indicating higher levels of each construct. In the present sample, internal consistency was good to excellent across assessment moments, with Cronbach’s alpha values ranging from 0.870 to 0.924 for cognitive anxiety, from 0.835 to 0.903 for somatic anxiety, and from 0.926 to 0.951 for self-confidence (Table 1).

**Table 1:** Inter-rater agreement for performance assessment across time points.

Subscale	*T*1 *α*	*T*2 *α*	*T*3 *α*
Cognitive anxiety	0.923	0.924	0.870
Somatic anxiety	0.903	0.849	0.835
Self-confidence	0.951	0.928	0.926

*α*, Cronbach’s alpha; CSAI-2, Competitive State Anxiety Inventory-2.

#### 2.3.3 Flow state

Athletes’ flow state was assessed using the Flow State Scale-2 (FSS-2; Jackson and Eklund, 2002), a self-report instrument designed to capture the subjective experience of flow during a specific performance episode. The FSS-2 reflects the multidimensional nature of flow, including challenge–skill balance, action-awareness merging, clear goals, unambiguous feedback, concentration on the task, sense of control, loss of self-consciousness, transformation of time, and autotelic experience. Items are rated on a Likert-type scale, with higher scores indicating a stronger flow experience. In the present study, the FSS-2 was administered after each competitive performance. Although the FSS-2 comprises multiple flow dimensions, the present study did not examine these dimensions separately. Instead, a global flow score was computed by averaging all items, with higher scores reflecting higher overall flow experience. Internal consistency for the global flow score was excellent across assessment moments, with Cronbach’s alpha values of 0.981, 0.953, and 0.943 for *T*1, *T*2, and *T*3, respectively.

#### 2.3.4 Perceived wellbeing

Athletes’ perceived wellbeing was assessed using the Hooper Index (Hooper and Mackinnon, 1995), a brief self-report monitoring tool commonly used in sport settings to assess athletes’ subjective recovery and psychophysiological state. The index comprises four single-item dimensions: sleep quality, fatigue, stress, and delayed onset muscle soreness (DOMS), each rated on a 7-point Likert-type scale. Higher scores indicate poorer perceived wellbeing, namely poorer sleep quality, greater fatigue, higher stress, and greater muscle soreness. In the present study, a global Hooper Index score was computed by summing the four items, with higher scores reflecting poorer overall perceived wellbeing/recovery. The four dimensions were also retained for descriptive purposes and, where relevant, complementary analyses. Given that each Hooper Index dimension is assessed by a single item and the global score represents a formative composite of conceptually related but distinct recovery indicators, internal consistency estimates such as Cronbach’s alpha were not computed.

#### 2.3.5 Performance assessment

Competitive performance was assessed at three different time points by three independent experts. For each athlete and at each time point, a performance score was provided by each expert. Inter-rater agreement was examined using the intraclass correlation coefficient (ICC), based on a two-way random-effects model with absolute agreement. Since the final performance score was computed as the average of the three expert ratings, ICC values for average measures were considered. The results indicated excellent inter-rater agreement across the three assessment moments, supporting the aggregation of the three expert ratings into a mean performance score for each time point (Table 2).

**Table 2:** Inter-rater agreement for performance assessment across time points.

Time point	*N* athletes	*N* raters	ICC average measures	95% CI	*F*	*p*
*T*1	80	3	0.956	[0.937, 0.971]	22.685	<0.001
*T*2	40	3	0.920	[0.865, 0.955]	12.322	<0.001
*T*3	20	3	0.916	[0.822, 0.964]	11.367	<0.001

ICC, intraclass correlation coefficient; CI, confidence interval. ICC values refer to average measures, based on a two-way random-effects model with absolute agreement.

Competitive performance was assessed *post hoc* through video analysis 1 week after the tournament. The rating grid was developed specifically for the purposes of this study and adapted to the technical and tactical demands of professional roller hockey. The experts were fully blinded to all athletes’ self-reported questionnaire responses and had no access to information regarding sport calling, perceived wellbeing/recovery, anxiety, self-confidence, or flow scores. The performance grid included four main indicators: technical execution, decision-making, defensive intensity, operationalized through ball recoveries, and collective contribution to team play. Ratings across these indicators were averaged to obtain a global competitive performance score for each athlete at each assessment moment.

### 2.4 Statistical analysis

All statistical analyses were conducted using IBM SPSS Statistics, version 31.0.2. Prior to hypothesis testing, data were screened for missing values, coding accuracy, and distributional properties. Descriptive statistics were computed for all study variables, including means, standard deviations, minimum and maximum values, skewness, and kurtosis. Distributional assumptions were inspected using skewness and kurtosis values. Following commonly used guidelines, absolute skewness values greater than 2 and absolute kurtosis values greater than 7 were considered indicative of substantial departures from normality, rather than strict cut-off criteria for normality (West et al., 1995; Kim, 2013). Internal consistency was assessed using Cronbach’s alpha for multi-item scales, whereas inter-rater agreement for performance ratings was examined using the intraclass correlation coefficient.

Pearson correlation coefficients were computed to examine preliminary bivariate associations among baseline sport calling, perceived wellbeing/recovery, competitive anxiety, self-confidence, flow, and performance. Given the longitudinal and unbalanced structure of the data, correlations were inspected by assessment moment. Same-time correlations were also examined to identify the most relevant associations among psychological states, recovery-related indicators, flow, and competitive performance before conducting the main analyses.

The first six hypotheses were tested using linear mixed models, an approach appropriate for longitudinal repeated-measures data, particularly when observations are unbalanced and participants contribute different numbers of measurements over time (Cnaan et al., 1997; Gueorguieva and Krystal, 2004). Separate models were estimated for competitive performance, flow, perceived wellbeing/recovery, self-confidence, cognitive anxiety, and somatic anxiety. In each model, time was specified as a fixed factor, baseline sport calling was included as the primary explanatory variable, and athlete identification was modelled as a random intercept to account for the non-independence of repeated observations within athletes, consistent with multilevel modelling principles (Snijders and Bosker, 2012). Restricted maximum likelihood estimation was used. Results were interpreted using tests of fixed effects, fixed-effect estimates, 95% confidence intervals, and pseudo-*R*^2^ indices. Marginal pseudo-*R*^2^ estimated variance explained by fixed effects, whereas conditional pseudo-*R*^2^ reflected variance explained by both fixed and random effects (Nakagawa and Schielzeth, 2013).

To complement these analyses, exploratory time-specific serial mediation models were estimated separately for each competitive moment using the PROCESS macro for SPSS, version 5.0. These models examined whether the pathway linking baseline sport calling to competitive performance through perceived wellbeing/recovery, self-confidence, and flow was consistent across tournament stages. Separate models were estimated for *T*1, *T*2, and *T*3 using PROCESS Model 6 (Hayes, 2022). Indirect effects were tested using 5,000 bootstrap samples and 95% bootstrap confidence intervals (Preacher and Hayes, 2008; Hayes, 2022). Because these analyses were conducted separately at each competitive moment, they did not model within-athlete change over time or the nested covariance structure of repeated observations. This distinction is important because mediation analyses with repeated-measures or hierarchically structured data require approaches capable of accounting for dependency among observations and variance at different levels of analysis (Bauer et al., 2006). Accordingly, the PROCESS analyses should not be interpreted as formal longitudinal multilevel mediation models or as evidence of a developmental causal mechanism. Instead, they were used as exploratory, time-specific indirect-effect analyses to examine whether the theoretically proposed pathway was observable at each competitive stage.

Given the progressive reduction in sample size imposed by the knockout structure of the tournament, the time-specific mediation analyses were interpreted with caution. This issue was especially relevant at *T*3, where only the finalists remained in the competition. The reduced number of observations at later stages limited statistical power, increased uncertainty around indirect-effect estimates, and may have compromised the stability of complex serial mediation pathways, particularly because mediation analyses are sensitive to sample size and require adequate power to detect indirect effects reliably (Schoemann et al., 2017). Accordingly, mediation findings at *T*3 were treated as exploratory rather than confirmatory.

Cognitive and somatic anxiety were not included in the serial mediation model because their associations with flow and performance were less consistent across competitive moments, particularly at the final stage. Although both dimensions are theoretically relevant in competitive sport, they did not show stable empirical associations with the subsequent variables in the proposed pathway across *T*1, *T*2, and *T*3. Since the aim was to test the same mediation sequence at each competitive moment, the final model prioritized variables that were consistently associated with one another across the tournament. This decision is consistent with mediation logic, in which indirect effects depend on the empirical links between the variables included in the pathway, particularly between each mediator and the subsequent outcome variable in the sequence (Preacher and Hayes, 2008; Hayes, 2022). In contrast, perceived wellbeing/recovery, self-confidence, flow, and performance showed more stable and theoretically coherent associations across the tournament, supporting their inclusion in the proposed serial pathway.

## 3 Results

### 3.1 Descriptive statistics

Descriptive statistics were computed for all study variables. Calling was assessed once at baseline and is presented in Table 3. Competitive state anxiety, flow, and performance were assessed across the three competitive moments and are presented in Table 4. Perceived wellbeing, including the global Hooper Index and its four indicators, is presented in Table 5. As expected, the number of valid observations decreased from *T*1 to *T*3 due to the knockout structure of the competition. Following commonly used guidelines, absolute skewness values below 2 and absolute kurtosis values below 7 were considered to indicate no substantial deviation from normality (West et al., 1995; Kim, 2013). Overall, all variables fell within these recommended thresholds, suggesting that the data did not present severe deviations from normality.

**Table 3:** Descriptive statistics for sport calling, competitive anxiety, self-confidence, flow, and performance across assessment moments.

Variable	Time point	*N*	Min	Max	*M*	SD	Skewness	Kurtosis
Calling	*T*0	80	3.20	8.70	6.92	1.15	−0.01	−0.39
Cognitive anxiety	*T*1	80	1.22	3.56	2.48	0.55	−0.21	−0.73
Cognitive anxiety	*T*2	40	1.33	4.00	2.37	0.52	0.51	1.05
Cognitive anxiety	*T*3	20	1.56	3.00	2.36	0.37	−0.35	0.31
Somatic anxiety	*T*1	80	1.33	3.89	2.55	0.49	0.14	−0.13
Somatic anxiety	*T*2	40	1.33	3.22	2.50	0.41	−0.51	0.58
Somatic anxiety	*T*3	20	2.00	3.22	2.74	0.38	−0.60	−0.82
Self-confidence	*T*1	80	1.11	3.89	2.53	0.73	0.19	−0.85
Self-confidence	*T*2	40	1.89	4.00	2.87	0.56	0.10	−0.69
Self-confidence	*T*3	20	1.89	4.00	2.98	0.55	0.18	−0.34
Flow	*T*1	80	0.94	4.72	2.76	1.11	0.08	−1.20
Flow	*T*2	40	1.86	5.00	3.64	0.96	−0.27	−1.09
Flow	*T*3	20	1.83	4.86	3.71	0.77	−0.66	0.49
Performance	*T*1	80	1.00	5.00	3.00	1.44	−0.02	−1.52
Performance	*T*2	40	1.33	5.00	3.64	1.09	−0.46	−0.85
Performance	*T*3	20	2.00	5.00	3.93	0.93	−0.72	−0.16

**Table 4:** Descriptive statistics for the global Hooper Index and its individual indicators across assessment moments.

Variable	Time point	*N*	Min	Max	*M*	SD	Skewness	Kurtosis
Global Hooper Index	*T*1	80	8.00	22.00	16.20	2.94	−0.25	−0.16
Global Hooper Index	*T*2	40	7.00	22.00	13.55	3.21	0.15	0.07
Global Hooper Index	*T*3	20	9.00	20.00	13.70	2.96	0.41	−0.27
Sleep quality	*T*1	80	2.00	6.00	4.11	0.83	0.20	0.01
Sleep quality	*T*2	40	2.00	5.00	3.70	0.82	0.04	−0.61
Sleep quality	*T*3	20	3.00	5.00	4.05	0.83	−0.10	−1.52
Stress	*T*1	80	1.00	7.00	4.05	1.24	0.03	−0.57
Stress	*T*2	40	2.00	6.00	3.70	0.94	0.26	−0.30
Stress	*T*3	20	2.00	5.00	3.60	0.94	−0.32	−0.58
Fatigue	*T*1	80	2.00	6.00	4.14	1.03	−0.21	−0.17
Fatigue	*T*2	40	1.00	6.00	3.00	1.32	0.07	−0.57
Fatigue	*T*3	20	1.00	6.00	2.75	1.16	0.76	2.20
Muscle soreness/DOMS	*T*1	80	2.00	6.00	3.90	0.79	0.02	−0.18
Muscle soreness/DOMS	*T*2	40	1.00	5.00	3.15	1.03	−0.17	−0.54
Muscle soreness/DOMS	*T*3	20	1.00	5.00	3.30	0.98	0.07	0.30

**Table 5:** Correlations between baseline calling and study variables across assessment moments.

Variable	*T*1	*T*2	*T*3
Cognitive anxiety	−0.669^***^	−0.320^*^	−0.021
Somatic anxiety	−0.504^***^	−0.347^*^	−0.398
Self-confidence	0.640^***^	0.536^***^	0.498^*^
Flow	0.771^***^	0.613^***^	0.740^***^
Performance	0.699^***^	0.613^***^	0.510^*^
Global Hooper Index	−0.781^***^	−0.829^***^	−0.656^**^
Sleep quality	−0.482^***^	−0.668^***^	−0.498^*^
Stress	−0.714^***^	−0.493^**^	−0.436
Fatigue	−0.658^***^	−0.771^***^	−0.653^**^
Muscle soreness/DOMS	−0.419^***^	−0.613^***^	−0.366

*T*1: *N* = 80; *T*2: *N* = 40; *T*3: *N* = 20. ^*^p < 0.05; ^**^p < 0.01; ^***^p < 0.001.

The baseline sport calling showed moderate mean levels, indicating variability in the extent to which athletes perceived their sport participation as a source of meaning and purpose. Across the competitive moments, self-confidence, flow, and performance tended to increase from *T*1 to *T*3, whereas the global Hooper Index decreased after the first competitive stage, suggesting more favorable perceived wellbeing/recovery among athletes who progressed in the tournament. Cognitive anxiety remained relatively stable across time points, while somatic anxiety showed a slight increase at *T*3.

### 3.2 Correlation analyses

After examining the descriptive statistics and distributional properties of the study variables, Pearson correlation analyses were conducted to explore the bivariate associations among the main constructs. Given the longitudinal and unbalanced structure of the data, correlations were inspected by assessment moment. Baseline sport calling was correlated with psychological states, perceived wellbeing/recovery, flow, performance, and the individual Hooper Index indicators across the three competitive moments. Same-time correlations were also examined to identify the most relevant associations among competitive anxiety, self-confidence, perceived recovery, flow, and performance before conducting the main analyses.

Baseline sport calling was positively associated with self-confidence, flow, and performance across the three competitive moments (Table 6). Calling was also negatively associated with the global Hooper Index and with several individual Hooper indicators, particularly stress and fatigue, indicating that athletes with stronger sport calling tended to report more favorable perceived recovery profiles. Negative associations were also observed between calling and both cognitive and somatic anxiety, especially at the earlier competitive stages, although these associations became weaker at *T*3.

**Table 6:** Same-time correlations among core psychological, recovery, and performance variables.

Association	*T*1	*T*2	*T*3
Cognitive anxiety – Flow	−0.800^***^	−0.605^***^	−0.308
Cognitive anxiety – Performance	−0.753^***^	−0.668^***^	−0.288
Somatic anxiety – Flow	−0.552^***^	−0.490^**^	−0.403
Somatic anxiety – Performance	−0.581^***^	−0.513^***^	−0.279
Self-confidence – Flow	0.879^***^	0.817^***^	0.798^***^
Self-confidence – Performance	0.857^***^	0.817^***^	0.915^***^
Flow – Performance	0.941^***^	0.937^***^	0.871^***^
Hooper Index – Cognitive anxiety	0.744^***^	0.593^***^	0.297
Hooper Index – Somatic anxiety	0.595^***^	0.346^*^	0.456^*^
Hooper Index – Self-confidence	−0.765^***^	−0.654^***^	−0.521^*^
Hooper Index – Flow	−0.878^***^	−0.756^***^	−0.831^***^
Hooper Index – Performance	−0.823^***^	−0.724^***^	−0.705^***^

*T*1: *N* = 80; *T*2: *N* = 40; *T*3: *N* = 20. Higher Hooper Index scores indicate poorer perceived wellbeing/recovery. ^*^p < 0.05; ^**^p < 0.01; ^***^p < 0.001.

Table 7 presents the same-time correlations among the core psychological, recovery, and performance variables. A consistent pattern emerged across the three assessment moments. Flow was strongly and positively associated with performance at all-time points, while self-confidence was also strongly related to both flow and performance. In contrast, cognitive and somatic anxiety tended to be negatively associated with flow and performance, particularly at *T*1 and *T*2. The global Hooper Index was positively associated with anxiety indicators and negatively associated with self-confidence, flow, and performance, suggesting that poorer perceived recovery was linked to less favorable psychological and performance-related outcomes.

**Table 7:** Same-time correlations between Hooper Index indicators, flow, and performance.

Hooper indicator	Flow *T*1	Perf. *T*1	Flow *T*2	Perf. *T*2	Flow *T*3	Perf. *T*3
Global Hooper Index	−0.878^***^	−0.823^***^	−0.756^***^	−0.724^***^	−0.831^***^	−0.705^***^
Sleep quality	−0.569^***^	−0.546^***^	−0.548^***^	−0.466^**^	−0.518^*^	−0.453^*^
Stress	−0.822^***^	−0.775^***^	−0.556^***^	−0.492^**^	−0.463^*^	−0.314
Fatigue	−0.721^***^	−0.645^***^	−0.722^***^	−0.719^***^	−0.815^***^	−0.666^**^
Muscle soreness/DOMS	−0.436^***^	−0.431^***^	−0.488^**^	−0.516^***^	−0.658^**^	−0.653^**^

Perf., performance. *T*1: *N* = 80; *T*2: *N* = 40; *T*3: *N* = 20. ^*^p < 0.05; ^**^p < 0.01; ^***^p < 0.001.

The global Hooper Index was consistently and negatively associated with both flow and performance across all competitive moments (Table 8). This indicates that poorer perceived recovery was related to lower flow and lower competitive performance. When the individual Hooper indicators were examined, fatigue showed one of the most consistent negative associations with both flow and performance across time points. Stress was also strongly associated with lower flow and performance, particularly at *T*1, whereas sleep quality and muscle soreness/DOMS showed moderate but generally consistent negative associations with performance-related outcomes.

**Table 8:** Linear mixed models testing the longitudinal effects of sport calling.

Hypothesis	Dependent variable	Fixed effect	*B*	SE	df	*t*	*p*	95% CI
H1	Performance	Calling	0.895	0.094	69.80	9.503	<0.001	[0.707, 1.083]
H2	Flow	Calling	0.752	0.068	73.40	11.059	<0.001	[0.616, 0.887]
H3	Hooper Index	Calling	−2.177	0.163	83.58	−13.383	<0.001	[−2.500, −1.853]
H4	Self-confidence	Calling	0.389	0.047	63.12	8.285	<0.001	[0.295, 0.482]
H5	Cognitive anxiety	Calling	−0.263	0.037	74.97	−7.113	<0.001	[−0.337, −0.190]
H6	Somatic anxiety	Calling	−0.193	0.032	136.00	−5.984	<0.001	[−0.257, −0.129]

SE, standard error; CI, confidence interval.

The correlational pattern suggests that sport calling, self-confidence, perceived recovery, and flow were meaningfully associated with competitive performance. In particular, the strong associations between flow and performance, together with the negative associations between poorer recovery and both flow and performance, provide preliminary support for examining these variables in the subsequent main analyses.

The magnitude of some same-time correlations should be interpreted with caution. Although the strong associations among self-confidence, flow, and performance are theoretically expected in competitive sport, as these constructs are proximal to psychological readiness and quality of execution, they may also indicate partial conceptual overlap. Importantly, the correlations exceeding 0.90 involved externally rated performance rather than athletes’ self-reported performance, which reduces, although does not eliminate, concerns regarding common method variance.

### 3.3 Hypothesis test

The first six hypotheses were tested using linear mixed models to account for the longitudinal and unbalanced repeated-measures structure of the data. In each model, time was specified as a fixed factor, baseline sport calling was included as the primary explanatory variable, and athlete identification was modelled as a random intercept to control for the non-independence of repeated observations within athletes.

Firstly, regarding the association between sport calling and competitive performance across the tournament the results showed a significant effect of time, *F*(2, 54.82) = 5.13, *p* = 0.009, indicating that performance ratings differed across competitive moments. More importantly, baseline sport calling was significantly and positively associated with competitive performance, *F*(1, 69.80) = 90.31, *p* < 0.001. As shown in Table 9, the fixed-effect estimate for calling was positive and significant, *B* = 0.895, SE = 0.094, *t*(69.80) = 9.50, *p* < 0.001, 95% CI [0.707, 1.083]. Thus, athletes reporting higher sport calling at baseline tended to receive higher performance ratings across the tournament, this result supporting H1. Moreover, the model explained a substantial proportion of variance in competitive performance, with a marginal pseudo-*R*^2^ of 0.473 and a conditional pseudo-*R*^2^ of 0.785 (Table 10). This suggests that fixed effects accounted for approximately 47.3% of the variance, whereas the full model, including the athlete-level random intercept, accounted for approximately 78.5% of the variance.

**Table 9:** Tests of fixed effects for the linear mixed models.

Hypothesis	Dependent variable	Effect	Num. df	Den. df	*F*	*p*
H1	Performance	Time	2	54.82	5.128	0.009
H1	Performance	Calling	1	69.80	90.307	<0.001
H2	Flow	Time	2	58.22	5.174	0.009
H2	Flow	Calling	1	73.40	122.295	<0.001
H3	Hooper Index	Time	2	85.87	7.702	<0.001
H3	Hooper Index	Calling	1	83.58	179.107	<0.001
H4	Self-confidence	Time	2	58.52	0.372	0.691
H4	Self-confidence	Calling	1	63.12	68.642	<0.001
H5	Cognitive anxiety	Time	2	82.62	1.428	0.246
H5	Cognitive anxiety	Calling	1	74.97	50.592	<0.001
H6	Somatic anxiety	Time	2	136.00	6.591	0.002
H6	Somatic anxiety	Calling	1	136.00	35.805	<0.001

Time was entered as a fixed factor with three assessment moments. Athlete identification was specified as a random intercept.

**Table 10:** Model fit and explained variance for the linear mixed models.

Hypothesis	Dependent variable	–2RLL	AIC	BIC	Marginal pseudo-*R*^2^	Conditional pseudo-*R*^2^
H1	Performance	378.824	382.824	388.648	0.473	0.785
H2	Flow	289.372	293.372	299.198	0.571	0.827
H3	Hooper Index	576.150	580.150	585.975	0.650	0.718
H4	Self-confidence	225.870	229.870	235.695	0.396	0.580
H5	Cognitive anxiety	180.599	184.599	190.425	0.290	0.381
H6	Somatic anxiety	157.371	161.371	167.196	0.228	0.228

–2RLL, −2 Restricted Log Likelihood; AIC, Akaike Information Criterion; BIC, Bayesian Information Criterion. Marginal pseudo-*R*^2^ reflects the variance explained by fixed effects; conditional pseudo-*R*^2^ reflects the variance explained by both fixed and random effects.

Regarding the relation between calling and flow across the tournament the results showed a significant effect of time, *F*(2, 58.22) = 5.17, *p* = 0.009, indicating that flow differed across competitive moments. And, baseline sport calling was a significantly and positively association with flow, *F*(1, 73.40) = 122.30, *p* < 0.001. As shown in Table 9, the fixed-effect estimate for calling was positive and significant, *B* = 0.752, SE = 0.068, *t*(73.40) = 11.06, *p* < 0.001, 95% CI [0.616, 0.887]. Thus, athletes reporting higher sport calling at baseline tended to experience higher levels of flow across the tournament, supporting H2. In addition, the model explained a substantial proportion of variance in flow, with a marginal pseudo-*R*^2^ of 0.571 and a conditional pseudo-*R*^2^ of 0.827 (Table 10). This indicates that fixed effects accounted for approximately 57.1% of the variance in flow, whereas the full model, including the athlete-level random intercept, accounted for approximately 82.7% of the variance.

The analysis of the relationship between baseline sport calling and perceived wellbeing/recovery (Hooper Index) across the tournament showed a significant effect of time, *F*(2, 85.87) = 7.70, *p* < 0.001, indicating that Hooper Index scores varied across competitive moments. Descriptively, scores decreased from *T*1 to *T*2 and remained lower at *T*3, suggesting more favorable perceived recovery after the first competitive stage. In the adjusted model, *T*2 showed significantly lower Hooper Index scores than *T*3, whereas *T*1 did not differ significantly from *T*3. More importantly, baseline sport calling was significantly and negatively association with Hooper Index scores, *F*(1, 83.58) = 179.11, *p* < 0.001. Because higher Hooper Index scores reflect poorer perceived wellbeing/recovery, this finding indicates that athletes with higher sport calling reported more favorable recovery profiles across the tournament, supporting H3. The model also explained a substantial proportion of variance, with a marginal pseudo-*R*^2^ of 0.650 and a conditional pseudo-*R*^2^ of 0.718 (Table 10).

Concerning H4 (Sport calling - athletes’ self-confidence) the results showed no significant effect of time, *F*(2, 58.52) = 0.37, *p* = 0.691, indicating that self-confidence did not differ significantly across competitive moments. However, baseline sport calling was significantly and positively associated with self-confidence, *F*(1, 63.12) = 68.64, *p* < 0.001. As shown in Table 9, the fixed-effect estimate for calling was positive and significant, *B* = 0.389, SE = 0.047, *t*(63.12) = 8.285, *p* < 0.001, 95% CI [0.295, 0.482]. Thus, athletes reporting higher sport calling at baseline tended to report higher self-confidence across the tournament, supporting H4. Moreover, the model explained a meaningful proportion of variance in self-confidence, with a marginal pseudo-*R*^2^ of 0.396 and a conditional pseudo-*R*^2^ of 0.580 (Table 10). This suggests that fixed effects accounted for approximately 39.6% of the variance in self-confidence, whereas the full model, including the athlete-level random intercept, accounted for approximately 58.0% of the variance.

With respect to the relationship between baseline sport calling and cognitive anxiety across the tournament, the results showed no significant effect of time, *F*(2, 82.62) = 1.43, *p* = 0.246, indicating that cognitive anxiety did not differ significantly across competitive moments. However, baseline sport calling was significantly and negatively associated with cognitive anxiety, *F*(1, 74.97) = 50.59, *p* < 0.001. As shown in Table 9, the fixed-effect estimate for calling was negative and significant, *B* = −0.263, SE = 0.037, *t*(74.97) = −7.113, *p* < 0.001, 95% CI [−0.337, −0.190]. Thus, athletes reporting higher sport calling at baseline tended to report lower cognitive anxiety across the tournament, supporting H5. In addition, the model explained a moderate proportion of variance in cognitive anxiety, with a marginal pseudo-*R*^2^ of 0.290 and a conditional pseudo-*R*^2^ of 0.381 (Table 10). This suggests that fixed effects accounted for approximately 29.0% of the variance in cognitive anxiety, whereas the full model, including the athlete-level random intercept, accounted for approximately 38.1% of the variance.

Regarding the relationship between baseline sport calling and somatic anxiety across the tournament, results showed a significant effect of time, *F*(2, 136) = 6.59, *p* = 0.002, indicating that somatic anxiety varied across competitive moments. Baseline sport calling was also significantly and negatively associated with somatic anxiety, *F*(1, 136) = 35.81, *p* < 0.001. As shown in Table 9, the fixed-effect estimate for calling was negative and significant, *B* = −0.193, SE = 0.032, *t*(136) = −5.984, *p* < 0.001, 95% CI [−0.257, −0.129]. Thus, athletes with higher baseline sport calling reported lower somatic anxiety throughout the tournament, supporting H6. The model explained 22.8% of the variance in somatic anxiety, with both marginal and conditional pseudo-*R*^2^ values equal to 0.228 (Table 10). The random intercept variance was estimated as zero, suggesting no additional between-athlete variance after accounting for time and calling.

Exploratory time-specific indirect-effect analyses were conducted to examine whether the association between baseline sport calling and competitive performance was statistically reflected in perceived wellbeing/recovery, self-confidence, and flow at each competitive moment. At *T*1, the total indirect effect of calling on performance through the proposed mediators was significant, Effect = 0.950, Boot 95% CI [0.775, 1.137]. The full serial indirect pathway from calling to performance through perceived wellbeing/recovery, self-confidence, and flow was also significant, Effect = 0.276, Boot 95% CI [0.162, 0.420]. The direct effect of calling on performance was not significant after including the mediators, *B* = −0.078, *p* = 0.328, suggesting that the association between calling and performance at *T*1 was primarily indirect.

At *T*2, a similar pattern emerged. The total indirect effect was significant, Effect = 0.582, Boot 95% CI [0.282, 0.855], and the full serial indirect effect through perceived wellbeing/recovery, self-confidence, and flow was also significant, Effect = 0.272, Boot 95% CI [0.081, 0.538]. The direct effect of calling on performance remained non-significant, *B* = 0.087, *p* = 0.435. These findings suggest that, at the second competitive stage, the association between calling and performance was also explained by recovery-related and optimal experience mechanisms.

At *T*3, the total indirect effect of calling on performance through the proposed mediators remained significant, Effect = 0.722, Boot 95% CI [0.380, 1.119]. However, the full serial indirect effect was no longer significant, Effect = 0.026, Boot 95% CI [−0.041, 0.137]. Thus, although the overall indirect association between calling and performance remained evident at the final stage, the complete serial pathway observed at *T*1 and *T*2 was not replicated at *T*3. Given the reduced sample size at *T*3 (*N* = 20), these findings should be interpreted as exploratory. The limited number of observations at the final stage reduced statistical power and may have increased the instability of indirect-effect estimates, particularly in a serial mediation model involving multiple intervening variables (Table 11).

**Table 11:** Indirect effects of baseline sport calling on competitive performance through perceived wellbeing/recovery, self-confidence, and flow.

Time point	Indirect effect	Effect	Boot SE	Boot 95% CI
*T*1	Total indirect effect	0.950	0.091	[0.775, 1.137]
*T*1	Calling → WB → Self-confidence → Flow → Performance	0.276	0.066	[0.162, 0.420]
*T*2	Total indirect effect	0.582	0.144	[0.282, 0.855]
*T*2	Calling → WB → Self-confidence → Flow → Performance	0.272	0.119	[0.081, 0.538]
*T*3	Total indirect effect	0.722	0.179	[0.380, 1.119]
*T*3	Calling → WB → Self-confidence → Flow → Performance	0.026	0.046	[−0.041, 0.137]

Overall, the exploratory time-specific mediation analyses provided partial support for H7 (The relationship between baseline sport calling and competitive performance will be serially mediated by perceived wellbeing/recovery, self-confidence, and flow. Specifically, athletes reporting higher sport calling before the competition are expected to report more favorable perceived recovery, which in turn is expected to be associated with higher self-confidence, leading to higher flow and, ultimately, better competitive performance). The full serial pathway was supported at *T*1 and *T*2, suggesting that athletes with higher sport calling tended to report more favorable perceived recovery, which was associated with higher self-confidence, greater flow, and better performance. At *T*3, the total indirect effect remained significant, but the full serial pathway was not statistically supported, indicating that the mechanism may be less stable at the final competitive stage or more difficult to detect due to the smaller sample size.

## 4 Discussion

The present study aimed to examine the role of sport calling in the competitive performance of professional roller hockey players, both directly and indirectly, by considering recovery-related, emotional, and experiential mechanisms across a knockout tournament. Drawing on a demands-resources perspective applied to sport, sport calling was conceptualized as a relatively stable psychological resource associated with how athletes interpret competitive demands, regulate their internal states, and respond to pressure in performance-related contexts. Overall, the findings support this formulation: sport calling was positively associated with performance, flow, and self-confidence, and was negatively associated with Hooper Index scores, cognitive anxiety, and somatic anxiety. In addition, the exploratory time-specific mediation analyses suggested that the association between calling and performance was consistent with an indirect pathway involving perceived recovery, self-confidence, and flow, particularly during the first two competitive moments.

The first relevant finding concerns the positive association between sport calling and competitive performance. Athletes who reported higher levels of calling before the tournament received higher performance ratings throughout the competition. This result is consistent with the vocational literature, which conceptualizes calling as an orientation with performance-related behavioral implications, rather than merely as a subjective experience of meaning (Duffy et al., 2018). This finding is also convergent with studies in work contexts showing that calling is related to performance, initiative, and active adaptation to task demands (Vianello et al., 2022; Yu et al., 2022), as well as with research suggesting that calling may foster more successful career trajectories (Abele and Spurk, 2009; Cho and Jiang, 2022). The present study extends this logic to the sport context, suggesting that the same orientation toward meaning, commitment, and identity is also associated with observable performance in a real competitive setting. Thus, sport calling appears as a motivational resource that is linked not only persistence intentions or subjective engagement, but also the quality of competitive execution.

The second finding showed that sport calling was positively associated with flow. This association is theoretically consistent with the definition of flow as an optimal psychological state marked by deep concentration, perceived control, absorption, automaticity, and intrinsically rewarding engagement (Csikszentmihalyi, 1990; Jackson and Marsh, 1996). The performance science literature has emphasized that flow emerges when there is alignment between challenge, perceived competence, attentional focus, and task meaning (Antonini Philippe et al., 2022; Biasutti and Philippe, 2023). Calling, in turn, involves a deep connection between identity, purpose, and activity. Accordingly, athletes with higher sport calling may be more predisposed to experience flow because they relate to competition in a more meaningful, engaged, and absorbing way. This finding contributes to the literature by suggesting that calling may be a relevant motivational antecedent of optimal functioning states in sport, bringing together two theoretical traditions that have been studied relatively separately: the literature on calling and the literature on flow.

The third finding showed that sport calling was positively associated with more favorable perceptions of wellbeing/recovery. This finding is particularly relevant because it helps position calling not only as a performance resource, but also as an adaptive resource. The sport recovery literature has moved away from a strictly physiological view, conceptualizing recovery as a multidimensional process involving bodily restoration, sleep, fatigue, stress, muscle soreness, psychological readiness, and adaptation to training and competition loads (Silva et al., 2026). Within this framework, recovery depends not only on the absence of physical fatigue, but also on how athletes interpret and regulate competitive demands. Convergently, Duffy et al. (2018) suggest that calling may promote meaning, satisfaction, and adaptive functioning when it is lived in a functional and self-regulated way. The present study suggests that this pattern may also be observable in professional roller hockey, particularly among male athletes competing in a high-pressure knockout tournament. Athletes with higher calling appear to report better perceived recovery, possibly because they are able to integrate fatigue, pressure, and discomfort within a more coherent and meaningful psychological framework.

The fourth set of findings showed that sport calling was associated with higher self-confidence and lower cognitive and somatic anxiety, consistent with literature on competitive anxiety and self-confidence. Self-confidence is considered a central psychological resource associated with perceived competence, control, adaptive coping, and better performance (Bandura, 1997; Craft et al., 2003; Woodman and Hardy, 2003). In contrast, cognitive anxiety involves worry and negative expectations, whereas somatic anxiety reflects perceived physiological activation associated with competition (Martens et al., 1990). The findings suggest that sport calling may be linked to a more adaptive pre-competitive experience by giving meaning to competition and strengthening the relationship between identity and action, thereby increasing perceived competence and reducing threat-based interpretations of pressure. Importantly, this suggests that calling may be understood not only as a distal motivational resource but also as an antecedent of psychological states proximal to competition. This aligns with the demands–resources perspective, which proposes that competitive demands may be interpreted as threats or challenges depending on available resources (Bakker and Demerouti, 2007; Balk et al., 2020).

The exploratory time-specific mediation results further refine this interpretation. The full serial pathway (sport calling → perceived recovery → self-confidence → flow → performance) was significant at *T*1 and *T*2, suggesting that the association between calling and performance is not only direct but also reflected in proximal psychological and recovery-related processes. Higher calling was associated with more positive perceptions of recovery, which were in turn associated with higher self-confidence; self-confidence was related to flow, and flow was strongly associated with better performance. This sequence aligns with literature positioning recovery as a foundation for psychophysiological readiness (Silva et al., 2026), self-confidence as a key indicator of psychological readiness (López-Hernández et al., 2026), and flow as an experiential state associated with fluid, automatic, and effective execution (Antonini Philippe et al., 2022; Swann et al., 2012). These findings are particularly relevant because they integrate different levels of performance explanation into a coherent process-based account of performance under pressure. Additionally these findings provide preliminary, time-specific evidence consistent with the idea that a distal motivational resource may be linked to competitive performance through variables increasingly proximal to the competitive moment.

However, the full serial pathway was not confirmed at *T*3, although the total indirect effect remained significant. This result requires careful interpretation. First, the reduced number of athletes in the final stage decreased the statistical power needed to detect complex indirect effects. Second, athletes who reach the final are likely to constitute a more homogeneous group in terms of competence, psychological resources, and adaptive capacity, which may reduce the variability required to identify statistical associations. Third, the final may represent a qualitatively distinct context in which other proximal determinants of performance, such as experience in decisive matches, collective strategy, leadership, decision-making under extreme pressure, or team dynamics, which were not assessed here, become more salient. Thus, the model appears to explain the early and intermediate stages of competition more effectively and may require adjustments to capture the psychological and contextual specificity of final-stage performance.

Although the exploratory mediation findings are theoretically coherent, the exceptionally high same-time correlations observed among perceived wellbeing/recovery, self-confidence, flow, and performance require a cautious interpretation of the proposed serial pathway and raise important questions about the conceptual distinctiveness of these constructs. Such high associations may indicate that, although the constructs are theoretically distinct, they partially overlap in the present competitive context. Perceived wellbeing/recovery, self-confidence, flow, and performance are all closely connected to athletes’ immediate readiness to compete and to the quality of their competitive functioning. However, this concern is less applicable to the correlations involving performance, as performance was not assessed through self-report but through ratings provided by three external experts who were blind to athletes’ questionnaire responses. This methodological feature reduces the likelihood that the strong associations between performance and the psychological variables are merely attributable to common method variance. Nevertheless, the magnitude of these correlations still suggests a close functional proximity between psychological readiness, optimal experience, and observable competitive execution, and therefore supports a cautious interpretation of the serial mediation model. This possible conceptual proximity appears particularly relevant in the association between self-confidence and flow. Self-confidence reflects athletes’ belief in their ability to respond successfully to competitive demands and is closely related to perceived competence, control, and adaptive coping (Bandura, 1997; Craft et al., 2003; Woodman and Hardy, 2003). Flow, in turn, refers to an optimal experiential state characterized by absorption, concentration, perceived control, action–awareness merging, and fluent execution (Csikszentmihalyi, 1990; Jackson and Marsh, 1996; Swann et al., 2012). Although these constructs are conceptually distinct, both include elements of perceived competence, control, and effective engagement with the task. Therefore, in high-pressure competitive contexts, self-confidence and flow may become empirically close because both reflect adaptive psychological functioning proximal to performance (Antonini Philippe et al., 2022; Koehn, 2013). A similar caution applies to the association between perceived wellbeing/recovery and flow. The Hooper Index assesses athletes’ subjective recovery status through sleep quality, fatigue, stress, and muscle soreness (Hooper and Mackinnon, 1995), whereas flow captures the quality of athletes’ optimal experience during performance. Nevertheless, recovery is increasingly understood as a multidimensional process involving physiological restoration, fatigue regulation, emotional balance, psychological readiness, and adaptation to training and competitive loads (Silva et al., 2026). In this sense, lower fatigue, lower stress, better sleep quality, and reduced muscle soreness may constitute the psychophysiological conditions that facilitate concentration, perceived control, absorption, and fluent action during competition. Thus, the strong association between the Hooper Index and flow may reflect not only a sequential pathway, in which better perceived recovery supports flow, but also partial conceptual proximity between perceived readiness and optimal experiential functioning. Taken together, this possible conceptual proximity has important implications for the validity of the serial mediation model. If perceived recovery, self-confidence, and flow are highly interrelated because they capture closely connected aspects of athletes’ immediate readiness and optimal functioning, the proposed pathway should not be interpreted as demonstrating a fully distinct, stepwise psychological mechanism. Rather, the findings should be understood as evidence that is consistent with the proposed process, but still preliminary regarding the separability and temporal ordering of the mediators. This is particularly relevant because the mediation analyses were time-specific and exploratory, rather than formal longitudinal multilevel mediation models capable of simultaneously modelling within-person change and between-person differences over time (Bauer et al., 2006; Hayes, 2022). Accordingly, although the observed indirect effects support the theoretical plausibility of the serial pathway, future studies should test the model using larger samples, temporally separated assessments, and longitudinal multilevel mediation designs to determine whether perceived recovery, self-confidence, and flow should be understood as distinct sequential mechanisms or as closely integrated components of a broader adaptive functioning state.

It is also important to discuss the role of anxiety in the mediation model. Although sport calling was significantly associated with lower cognitive and somatic anxiety, these variables were not included in the final serial model due to the lower consistency of their associations with flow and performance across competitive moments. This result does not reduce the relevance of anxiety within the pre-competitive profile, but suggests that, in this study, self-confidence constituted a more robust and stable mechanism. This interpretation is consistent with meta-analyses showing that self-confidence tends to show more consistent associations with performance than anxiety, particularly when anxiety is analyzed without considering its directional interpretation (Craft et al., 2003; Woodman and Hardy, 2003). Thus, anxiety appears as a relevant indicator of the pre-competitive response to calling, but not as a central mechanism in the explanatory pathway to performance in this specific design.

### 4.1 Main contributions

The present study offers two major theoretical contributions. First, it contributes to the literature on calling by extending this construct to the specific context of professional roller hockey. Although calling has been widely studied in vocational and occupational domains, its application to sport remains underdeveloped. By showing that sport calling was associated with performance, flow, perceived recovery, self-confidence, and competitive anxiety, this study demonstrates that calling can be conceptualized as a person–activity relationship that is relevant for understanding trajectories and experiences of sport excellence.

Second, it contributes to sport psychology by integrating calling, recovery, pre-competitive states, flow, and performance within a single model. Research on performance has often examined these variables separately. The present study proposes a more articulated interpretation, suggesting that performance may be understood in relation to a chain of processes: a motivational-identity resource linked to perceived recovery, which is associated with self-confidence, which is in turn related to flow, and flow was positively associated with better performance. This approach brings sport psychology research closer to a genuinely process-based and ecological account of performance.

From a practical standpoint, the results suggest that coaches, sport psychologists, and performance staff should consider the meaning athletes attribute to their sport as a relevant resource for both performance and wellbeing. Interventions focused on purpose, athletic identity, self-regulation, and balance between commitment and recovery may help athletes use calling as a functional resource, preventing dedication from becoming overinvestment or strain.

### 4.2 Limitations and future research

Despite the theoretical, methodological, and applied contributions of this study, some limitations should be acknowledged.

First, although conducted with professional athletes in a real competitive context, the findings are restricted to professional male roller hockey players from a single national context and assessed within a specific knockout tournament format. The results may be particularly informative for understanding performance-related psychological processes in roller hockey sport characterized by high intensity, rapid decision-making, technical-tactical demands, and repeated exposure to competitive pressure. Future research should therefore replicate the proposed model in this sporting context, including professional female players.

A second limitation concerns the *T*3 results, which should be considered exploratory due to the small sample size (20). Although the naturalistic design strengthened ecological validity by examining performance under real competitive conditions, it produced a progressively unbalanced data structure, with the sample decreasing from 80 athletes at *T*1 to 40 at *T*2 and 20 at *T*3. This reduction has important statistical implications. Smaller sample sizes reduce statistical power, increase uncertainty around parameter estimates, widen or destabilize confidence intervals, and limit the robustness of complex indirect-effect models. These constraints are particularly relevant for the *T*3 mediation analysis, which involved only the finalists and should therefore be interpreted as exploratory rather than confirmatory. Future studies should test the model in competitions with larger and more stable samples or through extended longitudinal designs, such as across an entire season.

A third limitation relates to the use of self-report measures to assess recovery, anxiety, self-confidence, and flow. Although these measures are appropriate for capturing subjective states and internal psychological experiences, they may be subject to social desirability bias, response fatigue, or the influence of competitive outcomes. Future studies should combine self-reports with objective and physiological indicators, such as actigraphy, objective sleep quality and duration, heart rate variability, internal and external load, fatigue markers, match statistics, and behavioral indicators of performance.

A fourth limitation concerns the mediation analysis. The mediation models were estimated separately by competitive moment and should be interpreted as complementary analyses, rather than as formal longitudinal multilevel mediation models. This option made it possible to explore the temporal consistency of the proposed mechanism across different tournament stages but does not fully capture the hierarchical and dynamic structure of the data. Future studies with larger samples and longitudinal multilevel mediation models are needed to confirm the proposed mechanism, capable of simultaneously analysing within-person and between-person variation, as well as more robust temporal relationships among calling, recovery, psychological states, flow, and performance.

### 4.3 Conclusion

This study suggests that sport calling constitutes a relevant psychological resource for understanding performance among male professional roller hockey players competing in a high-pressure knockout tournament. The findings indicate that athletes who experience their sport as a source of meaning, identity, and purpose present better performance, higher flow, better perceived recovery, greater self-confidence, and lower competitive anxiety. More importantly, the exploratory time-specific mediation analyses suggested that the association between calling and performance was not only direct but was also consistent with an indirect pathway: in the early and intermediate stages of the tournament, this association was consistent with an adaptive sequence involving perceived recovery, self-confidence, and flow. Although this full pathway was not confirmed in the final, the findings reinforce the importance of understanding athletic performance as a multidimensional phenomenon in which motivational, recovery-related, emotional, and experiential resources interact to sustain performance under pressure. In this way, the study positions sport calling as a promising construct in sport psychology, with relevant implications for research, assessment, and intervention in high-performance contexts.

## Data Availability

The datasets presented in this study can be found in online repositories. The names of the repository/repositories and accession number(s) can be found below: https://doi.org/10.5281/zenodo.20344783.
